# Developing Social Media-Based Suicide Prevention Messages in Partnership With Young People: Exploratory Study

**DOI:** 10.2196/mental.7847

**Published:** 2017-10-04

**Authors:** Jo Robinson, Eleanor Bailey, Sarah Hetrick, Steve Paix, Matt O'Donnell, Georgina Cox, Maria Ftanou, Jaelea Skehan

**Affiliations:** ^1^ Orygen, The National Centre of Excellence in Youth Mental Health Centre for Youth Mental Health University of Melbourne Parkville Australia; ^2^ String Theory Creative Melbourne Australia; ^3^ Melbourne School of Population and Global Health University of Melbourne Melbourne Australia; ^4^ Hunter Institute of Mental Health Newcastle Australia

**Keywords:** suicide, suicidal ideation, social media, youth, adolescents, mass media

## Abstract

**Background:**

Social media is increasingly being used by young people for health-related issues, including communicating about suicide. Due to the concerns about causing distress or inducing suicidal thoughts or behaviors, to date young people neither have been engaged in the development of social media–based suicide prevention interventions nor have interventions focused on educating young people about safe ways to communicate about suicide online. Given the potential that social media holds to deliver messages to vast numbers of people across space and time and the fact that young people often prefer to seek help from their friends and peers, safely educating and engaging young people to develop suicide prevention messages that can be delivered via social media is an obvious next step.

**Objectives:**

The objectives of this study were to (1) provide education to a small number of secondary school students about safe ways to communicate about suicide via social media; (2) engage the same young people in the development of a suite of social media–based suicide prevention multimedia messages; (3) assess the impact of this on participants; and (4) assess the acceptability and safety of the messages developed.

**Methods:**

This study involved two phases. In phase 1, 20 participants recruited from two schools took part in an 8- to 10-week program during which they were provided with psychoeducation about mental health and suicide, including how to talk safely about suicide online, and they were then supported to design and develop their own media messages. These participants completed an evaluation questionnaire at the conclusion of the program. In phase 2, a larger group of participants (n=69), recruited via an opt-in process, viewed the media messages and completed a short questionnaire about each one.

**Results:**

Participants in phase 1 enjoyed the program and reported that they learned new skills, such as how to talk safely about suicide online, and felt more able to provide emotional support to others (16/20, 80%). No participants reported that the program made them feel suicidal. Participants in phase 2 generally rated the media messages as safe and acceptable, although some messages were rated more highly than others.

**Conclusions:**

This study suggests that young people can be safely engaged in developing suicide prevention messages, which can be disseminated via social media. Engaging young people in this process may improve the traction that such campaigns will have with other young people. The study also suggests that educating young people regarding how to talk safely about suicide online has multiple benefits and is not associated with distress. Overall, these findings pave the way for new approaches to prevent suicide among young people.

## Introduction

### Suicide and Young People

Suicide is the leading cause of death among young people worldwide, including Australia [1,2]. It accounts for one-third of all deaths in Australians younger than 25 years and, despite decades of government attention and some improvement in youth suicide rates between 1997 and 2012, rates have increased again in the recent years [1]. In addition, many more young people make a suicide attempt and even more live with suicidal feelings [3,4].

### The Relationship Between Suicide and the Media

Certain types of media reporting on suicide have been linked to an increase in suicide deaths [5]. This is thought to be the result of contagion, whereby the suicide of one individual may lead another person (particularly someone who identifies with the deceased) to take their own life; young people are thought to be particularly susceptible to this process [6]. For this reason, the role of the media in suicide prevention has long been recognized. Indeed, the World Health Organization [2] states that working with media on the responsible reporting of suicide is an evidence-based suicide prevention strategy that should form part of national suicide prevention approaches. In response to this and to research that suggests an association between media reporting of suicide and suicidal behavior, several countries, including Australia, have developed media guidelines that advocate for responsible and sensitive reporting and portrayal of suicide [5,7-10]. However, the increasing popularity of social media platforms such as Facebook, Instagram, YouTube, and Snapchat, in particular among young people, has presented a new set of opportunities and challenges for suicide prevention. For example, challenges include the potential for further contagion and the spreading of information about suicide methods [11]. A further challenge arises from the fact that because young people are now the creators of their own content, strategies that engage media professionals to safely and responsibly report on suicide may have limited impact on other communication channels such as social media [12].

Despite these challenges, there are also potential benefits that arise from the reach, acceptability, and cost-effectiveness of interventions delivered via social media platforms [13,14]. To date, however, there has been limited evaluation of social media–based interventions, largely because of methodological challenges associated with the fast-moving, amorphous, and anonymous nature of these platforms [13-17]. As a result, new approaches to prevent youth suicide and to evaluate youth suicide prevention efforts are required [18]. One such approach could involve educating and supporting users directly about safe and unsafe ways to communicate about suicide via social media; to the best of our knowledge, this approach has not been tested.

In addition to strategies that influence the news and entertainment media, the delivery of population-wide suicide prevention media campaigns has also gained attention as a possible effective strategy. Limited evidence exists for the effectiveness of these campaigns to change behavior; however, they have been shown to improve outcomes such as knowledge and awareness of suicide, attitudes toward suicide and help-seeking, and may have the potential to reduce suicide rates [19-25]. To date, however, limited evidence exists regarding the impact of such campaigns, specifically on young people, and few campaigns, if any, focus purely on social media or actively involve young people in their design and implementation.

Participatory design processes are critical to ensure that the views and preferences of end users are accounted for, which in turn is likely to enhance uptake and engagement [26]. These processes may be especially important in the development of suicide prevention materials, given that young people are more likely to seek help from their friends than professionals [27,28]. Researchers have so far successfully engaged young people in the development of computerized cognitive behavioral therapy programs for depression and psychosis [29,30]. To date, however, no studies have reported on the development of media messaging on suicide prevention using participatory design processes.

### Study Aims

To address this gap, this exploratory study aimed at the following:

1. To engage small groups of young people (up to 10 per group) in the development of a suite of suicide prevention media messages that can be delivered to other young people via social media platforms;
2. To assess the impact of participating in the program on participants’ knowledge of mental health issues and suicide, their ability to talk safely about suicide both online and offline, and any potential iatrogenic effects; and
3. To evaluate the acceptability, efficacy, and safety of the media messages developed.

## Methods

### Study Design

The methodology comprised two phases. Phase 1 examined the perceived impact of participating in the program on participants. This was assessed by a specifically designed questionnaire administered at the end of the program. Phase 2 examined the perceived acceptability of the media messages by a wider group of students. To evaluate this, brief Web-based questionnaires were administered immediately after viewing the messages.

### Setting

The study was conducted by researchers from Orygen, The National Centre of Excellence in Youth Mental Health, in partnership with String Theory Creative, a creative and digital communications agency. String Theory Creative provided education to participants on using digital media (including how to edit short films) and also provided assistance in producing the media messages (eg, balancing sound and editing footage where necessary). Other partners were the University of Melbourne and the Hunter Institute of Mental Health, who developed the Mindframe resources for media professionals reporting on suicide [9].

### Phase 1: Development of Media Messages

#### Participants

Participants were students from two secondary schools in Melbourne, Australia. One school was a coeducational vocational college and the other was a high-performing all-boys school. Recruitment into the study was different for each school. For School 1, participants were students from two senior business marketing classes, and the program was integrated into the curriculum. Students in these classes who did not want to participate were given alternative work to complete in a different location. For School 2, the study was advertised on the school news feed (accessed by both parents and students) and was also promoted by the school counselor to some students. Students at School 2 self-selected into the study.

#### Measures

A Web-based survey was developed consisting of items specifically developed for this study (Multimedia Appendix 1). Participants were asked for their age and gender, as well as 6 questions about their personal experience with suicidal thoughts, self-harm, mental health problems, helping a friend with mental health problems or suicidal thoughts, and bereavement by suicide. A total of 18 questions were used to assess participants’ views on the project, including whether they had learned new skills with regard to communicating about suicide; whether they found it enjoyable, worthwhile, or upsetting; and whether they would recommend it to a friend.

#### Procedure

The program comprised eight sessions delivered over an 8- to 10-week period. Each session lasted approximately 2 hours and was delivered by a research assistant (EB), with support from senior researchers (JR and SH) and a staff member from String Theory Creative (SP). The sessions were delivered in school premises during school hours and at least one school staff member was either present or nearby during the sessions. No homework was set; however, students were encouraged to work on their media messages in their own time if they did not have sufficient time during the sessions. Table 1 presents the structure of sessions.

#### Intervention

The intervention took the form of the development of a suite of media messages made by young people for other young people. No limits were imposed on either the format or content of the media messages. In developing their media messages, participants were encouraged, however, to use their existing resources. For example, they were asked to record video footage using their mobile phones or tablets and edit it using software already installed on their school computers. One researcher (SP) with extensive experience in recording and editing videos was available to provide assistance if required.

**Table 1:** Structure of sessions.

Session	Description
1	Meet participants and introduce project;
	Discuss goals for the program and set boundaries; and
	Show some examples of social media being used for suicide prevention.
2	Provide psychoeducation about mental health and suicide, including information about: the prevalence of mental health problems, including depression and anxiety, and suicide and suicidal behavior; signs and symptoms of mental health problems; and warning signs and risk factors for suicide.
3	Provide psychoeducation about safe ways of communicating about suicide based on resources such as the Mindframe media guidelines [9]; and
	Provide psychoeducation about how to help a friend who might be thinking about suicide based on resources from mental health organizations such as SANE, headspace, and beyondblue.
4	Brainstorm with students about ideas for their media messages and decide on concepts; and
	Provide education about designing and creating multimedia content.
5-7	Development of media messages.
8	Presentation day for media messages; and
	Participants complete questionnaires.

Two researchers (JR and EB) oversaw the content of the media messages, giving feedback and advice where necessary to ensure their safety and appropriateness. For the most part, participants created their media messages during the sessions; however, some also worked on them in their own time.

A closed Facebook group was set up for each group of study participants so that participants could communicate about the project outside the school-based sessions, and the research team could share relevant information, such as links to useful websites, with the students. The Facebook groups were moderated daily by a research assistant (EB) and deleted on completion of the program.

### Phase 2: Evaluation of Media Messages

#### Participants

Participants were students from the same two secondary schools described above. In both schools, the study was advertised to all year 11 and 12 students (aged 16-18 years). Students who provided informed consent were eligible to participate in the evaluation.

#### Measures

A survey was specifically designed for this purpose (Multimedia Appendix 2). It contained 1 item about participants’ age and 3 items about the experience of suicidal thoughts (lifetime, within the last 4 months, right now). It also contained 9 questions for each of the media messages being evaluated, which did not change between evaluations. Questions 1 and 2 required participants to indicate how helpful they thought the message would be for someone who is experiencing thoughts of suicide or wants to help somebody else experiencing suicidal thoughts. Questions 3 and 4 required participants to indicate how likely they were, after viewing the intervention, to seek help for suicidal thoughts or help somebody else experiencing suicidal thoughts. Questions 5 and 6 required participants to rate their mood before and after viewing the media message using a 7-point modified faces pain rating scale. Questions 7 and 8 required participants to specify their thoughts about the content and format of the media message. Finally, Question 9 asked whether participants would share the message with others.

#### Procedure

Participants viewed the media messages and subsequently completed the Web-based survey at school in the presence of a research assistant (EB). EB received an automated email if any participant indicated that they had experienced recent or current suicidal thoughts. These participants were responded to as per the safety protocol (see below).

### Data Analysis

Qualtrics survey software was used to collect all data. Simple frequencies and percentages were calculated for each set of response options.

### Ethics and Safety

The evaluation received approval from the Melbourne University Human Research and Ethics Committee (ID 1442942). All students provided written consent to take part in the study. In the case of students aged less than 18 years, consent was also obtained from a parent or guardian.

A comprehensive safety protocol was developed to ensure that any participant determined to be at risk at any stage of the process would be recognized and responded to appropriately. This included responses to (1) survey items pertaining to suicidality; (2) any student who became distressed during one of the school-based sessions; and (3) any student who indicated distress on the Facebook group. In all cases, the protocol required that if a student was identified as being potentially at risk, the RA would conduct a risk assessment and, if required, refer the student to the school well-being team for follow-up.

## Results

### Phase 1: Development of Media Messages

#### Participants

A total of 26 students took part in the project, of whom 20 completed the questionnaires. The 6 participants who did not complete questionnaires had left either the school or class during the study period.

Among these students, 80% (16/20) were male, and the mean age was 17.1 years (standard deviation 1.69). At baseline, 45% (9/20) of the students had experienced a mental health problem in their lifetime, 55% (11/20) had experienced suicidal thoughts or feelings, 20% (4/20) had engaged in self-harm, 80% (16/20) had supported a friend experiencing mental health problems, and 70% (14/20) had supported a friend experiencing suicidal thoughts. In addition, 30% (6/20) of the students said that somebody close to them had died by suicide.

#### Messages Developed

A total of 8 media messages were produced. Of these, 7 were short videos (ranging from 30 s to 3 min in duration) and one was a series of 4 images designed to be used on a platform such as Instagram or Snapchat. A description and screenshot for each media message is shown in Multimedia Appendix 3. All the media messages contained contact information for helplines or services.

#### Project Evaluation

Participants’ responses to the project evaluation questions are displayed in Tables 2 and 3. Tables 2 and 3 contain participants’ responses to the items about knowledge or skills gained and about project safety and acceptability, respectively.

In general, participants reported that they had gained new skills and abilities. In particular, most participants (16/20, 80%) felt more able to talk about suicide both generally and online, and the same number felt better able to provide emotional support to others.

**Table 2:** Skills or knowledge gained through Safe Conversations.

Variable	Agree or strongly agree, n (%)	Neutral, n (%)	Disagree or strongly disagree, n (%)
Participating in this project has helped me to develop new skills.	18 (90)	1 (5)	1 (5)
As a result of this project, I have a better understanding of how to talk about suicide safely online.	16 (80)	3 (15)	1 (5)
As a result of this project, I have a better understanding of how to talk about suicide generally.	16 (80)	4 (20)	0
The project has helped to improve my self-confidence.	9 (45)	7 (35)	4 (20)
The project has helped me to develop my leadership and mentoring skills.	10 (50)	9 (45)	1 (5)
The project has helped me to further develop my interpersonal skills.	12 (60)	7 (35)	1 (5)
The project has helped me to further develop my communication skills.	14 (70)	5 (25)	1 (5)
As a result of participating, I feel more able to provide emotional support to others.	16 (80)	4 (20)	0
As a result of participating, I feel more able to educate others about cyber safety.	14 (70)	6 (30)	0

**Table 3:** Acceptability and safety of Safe Conversations project.

Variable	Agree or strongly agree, n (%)	Neutral, n (%)	Disagree or strongly disagree, n (%)
The Safe Conversations project was enjoyable.	16 (80)	4 (20)	0
The Safe Conversations project was helpful.	17 (85)	3 (15)	0
The Safe Conversations project made me feel upset.	1 (5)	4 (20)	15 (75)
The Safe Conversations project made me feel suicidal.	0	3 (15)	17 (85)
The Safe Conversations project was boring.	2 (10)	3 (15)	15 (75)
The Safe Conversations project took up too much of my time.	0	6 (30)	14 (70)
I found participating in the Safe Conversations project stressful.	0	4 (20)	16 (80)
I feel motivated after participating in the Safe Conversations project.	10 (50)	5 (25)	2 (10)
The Safe Conversations project was worthwhile.	15 (75)	4 (20)	1 (5)

**Table 4:** Participants’ responses to the evaluation questions.

Questionnaire item	Beach video, n (%)	Vox pop, n (%)	Signs video, n (%)	Post-it-note video, n (%)	Suicide can’t be reversed, n (%)	Letter video, n (%)	Mask video, n (%)	Series of 4 images, n (%)
Helpful or extremely helpful for suicidal person	16 (80)	7 (39)	8 (42)	30 (71)	33 (79)	24 (57)	8 (19)	7 (70)
Helpful or extremely helpful for someone wanting to help a suicidal person	13 (65)	8 (45)	7 (37)	35 (83)	36 (86)	24 (57)	12 (29)	6 (60)
Less likely to seek help	1 (5)	0	4 (21)	0	2 (5)	0	16 (38)	0
More likely to seek help	6 (6)	2 (11)	4 (21)	15 (36)	19 (45)	15 (36)	3 (7)	5 (50)
More likely to help a friend	10 (50)	6 (33)	6 (32)	23 (55)	25 (60)	29 (69)	7 (17)	4 (40)
Mood decreased after viewing	1 (5)	1 (6)	1 (5)	8 (19)	19 (45)	29 (69)	11 (26)	0
Likes or really likes the format	11 (55)	5 (28)	9 (47)	21 (50)	32 (76)	23 (55)	12 (29)	9 (90)
Likes or really likes the content	12 (60)	12 (67)	8 (42)	29 (69)	30 (71)	30 (71)	12 (29)	7 (70)
Would share with others	8 (40)	10 (56)	6 (32)	22 (52)	28 (67)	20 (48)	9 (21)	5 (50)
Total N	20	18	19	42	42	42	42	10

Most participants (16/20, 80%) enjoyed participating in Safe Conversations; 17 (85%) thought it was helpful, and 15 (75%) thought it was worthwhile. No participants thought that the program was stressful or time consuming, and no participants reported that the program made them feel suicidal (although one participant said that the program made them feel upset). In addition, no participants became visibly distressed during any of the sessions. Likewise, there were no occasions where participants communicated that they were distressed or suicidal using the Facebook groups. Finally, 19 (95%) participants said that they would recommend the project to a friend.

### Phase 2: Evaluation of Media Messages

#### Participants

A total of 69 participants took part in the evaluation, including 18 from School 1 and 51 from School 2. Of these, 10 participants were students from School 2, who participated in phase 1 of the project and had recently completed the phase 1 questionnaire; as such, they did not provide any additional demographic or suicide risk information.

The mean age of the remaining participants (n=59) was 16.4 years. Of these, 25 (42%) reported that they had experienced suicidal thoughts or feelings at some point in their lives, 5 (9%) reported that they had experienced these feelings in the past 4 weeks, and 1 (2%) reported that they were currently experiencing these feelings.

#### Evaluation Data

Responses of the participants to each of the media messages described above are shown in Table 4. The total number of participants who evaluated each media message differed because participants at School 1 were only required to evaluate at least two media messages because of time constraints. As such, not all participants evaluated all media messages. Moreover, participants in phase 1 at School 2 evaluated the media messages developed by School 1.

The media messages varied in terms of how helpful they were perceived to be for a person experiencing suicidal thoughts. The “beach” video was rated as the most helpful (rated as “helpful” or “very helpful” by 80% of participants [16/20]), followed by the “suicide can’t be reversed” video (33/42, 79%), the “post-it-note” video (30/42, 71%), and the series of 4 images (7/10, 70%). The majority were rated as being more helpful for someone who wants to help a suicidal person than for a suicidal person themselves, although there were three exceptions (beach video, signs video, and image series). The “suicide can’t be reversed” video was rated as most helpful for someone who wants to help somebody else having thoughts of suicide (36/42, 86%).

The format that participants liked the most was the series of 4 images (90% of participants [9/10] either “liked it” or “really liked it”); this was the only media message that took the format of a static image rather than a video. The majority of participants generally liked the content of the media messages, with the exception of the “mask” and the “signs” videos that were liked by only 29% (12/42) and 42% (8/19) of participants, respectively. Approximately 50% of the participants were likely to share the media messages with others.

Most of the media messages were *not* associated with a decrease in help-seeking intentions, although 38% of participants (16/42) reported a decrease in their likelihood of seeking help after seeing the “mask” video. Although the majority of participants indicated that their help-seeking intentions remained the same, all media messages were associated with increased likelihood of seeking help in at least some participants (ranging from 7% to 50%). Additionally, all media messages, with the exception of the image series, were more highly associated with increased likelihood of helping others than they were with increased likelihood of helping oneself.

Most of the media messages were not associated with a decrease in mood, including among those participants who had experienced recent suicidal ideation. However, almost 70% of the sample (29/42) reported that their mood decreased after viewing the “letter” video, including 2 of the 5 participants who had experienced recent suicidal ideation. Despite this, 71% (30/42) reported that they liked the content and 57% (24/42) thought it would be helpful for a suicidal person.

## Discussion

### Principal Findings

This exploratory study examined the feasibility, safety, and impact of engaging young people in the development of suicide prevention media messages that can be delivered to other young people via social media. In total, 8 media messages were produced, including 7 short films and one series of static images. Overall, students found the program to be useful and worthwhile. The majority of students reported that they felt better able to communicate safely about suicide, including online. In addition, they reported to feel better able to provide emotional support to others and educate others about staying safe online. Notably, no students felt suicidal as a result of taking part in the program, and the majority reported the program to be enjoyable, helpful, and worthwhile.

In general, the media messages developed were found to be both safe and acceptable, although some more so than others. For example, the format that participants liked the most was the series of 4 images; this was the only message that adopted a static format. This message was also rated by the most participants as increasing their likelihood of seeking help and was the only one that was not associated with any decrease in participants’ mood. Another media message of note was the “letter” video, which was most strongly associated with a decrease in mood. Despite this, most participants reported that they liked the content, over 50% of the participants thought it would be helpful for a suicidal person, and it was not associated with decreased likelihood of seeking help. Also of note were the participants’ ratings of the “mask” video, as it was the only message designed to be humorous. This was arguably the least acceptable message, with less than one-third of participants reporting that they liked the format or content and less than 20% thinking that it would be helpful for a suicidal person. Moreover, 38% of participants reported a decreased likelihood of seeking help after viewing it; this was by far the highest percentage in this category. Finally, participants rated all but one of the media messages as more likely to improve their intentions to *help others* than their intentions to *seek help for themselves*. This suggests that media messages may have the potential to influence viewers to talk to people who they are worried about. Overall, these data provide some indication about the type of medium and content that appears to be most appealing to young people and the potential impact they may have on the likelihood of young people helping themselves and others. It also indicates that just because a media message may lead to a decrease in mood in some viewers does not mean that it is perceived to be unhelpful. It is hoped that these findings may help in the development of future suicide prevention campaigns.

### Limitations

First, this was a small study conducted in two secondary schools in Melbourne, Australia. One school was an all-boys school and the other was a vocational secondary college. As such, the results obtained in this study may not necessarily be generalizable to other school settings. It is also worth noting that a significant proportion of the sample reported previously experiencing either a mental health problem, suicidal feelings and/or supporting a friend with suicidal feelings, which may also impact upon the generalizability of the study findings.

Second, this was a simple posttest study that assessed participants’ perceptions using a simple survey administered at one time point. This was intentional based on the exploratory nature of the project, but it does impact on the robustness of the findings.

Third, we did not have the scope to examine the reach and impact of the media messages developed beyond what is described above. As such **,** the findings with regard to the potential impact of the media messages outside the school context must be interpreted with caution. On a related note, we were not able to examine how the messages were perceived by mental health professionals or suicide prevention experts. There is a risk that, although they were rated favorably by young people, professionals may not consider think them as safe or acceptable; this should be the focus of future studies of this nature.

### Comparison With Prior Work

Notwithstanding the limitations, this novel study has implications regarding the ways in which social media can be used in suicide prevention and how young people can be engaged in this process. It also builds on previous work in numerous ways.

The ways in which suicide is discussed in the media have long been considered to have an impact on suicide-related behaviors, in particular, among vulnerable individuals [31,32]. As noted above, historically, a key and accepted approach to address this has been through the development and active dissemination of guidelines for media professionals using traditional media outlets [9,10,33]. It has been suggested that similar guidelines should be developed for Web-based platforms [12]. Although some such guidelines exist, such as those developed by the International Association for Suicide Prevention to assist bloggers reporting on suicide [34] and advice developed for social media content under the Mindframe National Media Initiative in Australia [35], their impact on the ways in which suicide is discussed online has not been tested and may be limited. For example, without targeting young people directly, these guidelines are unlikely to impact the way young people discuss suicide with their peers using social media platforms, where consumers, not professionals, create their own content. As such, it is acknowledged that a different approach is required [36]. The approach taken in this study of educating young people regarding safe ways to communicate about suicide and then supporting them to develop their own media messages appears to be promising. This was not only the case in terms of participants’ perceived ability to communicate more safely about suicide but also their perceived ability to support others expressing emotional distress, both online and offline.

Concerns have been expressed on the safety of communicating about suicide via social media platforms [14-16] and about the safety of delivering suicide prevention education to young people in a classroom setting [37]. The fact that no immediate adverse effects were reported by the students who participated in this study, however, suggests that both of these strategies can be implemented safely, as long as care is taken to ensure that participants are monitored for signs of distress and responded to appropriately if required. This supports our recent research indicating that educating high school students about suicide prevention is both safe and acceptable [38] and that social media holds potential for the delivery of suicide prevention messages [13,39].

It is well documented that young people prefer to talk to their friends and peers than health professionals about emotional problems [27,40]. In addition, one aspect of social media valued by young people is its ability to connect them with others who have had similar experiences in a nonjudgmental and more egalitarian way [13,41]. Thus, it stands to reason that social media messages that have been developed by young people may well be more acceptable to other young people than those developed by adults. This study provides evidence that young people can successfully be engaged in this process.

The type of media messages developed here could readily be delivered via platforms such as YouTube, Snapchat, and Instagram either as stand-alone messages or as part of a large-scale suicide prevention campaign. Despite recent interest in the development of suicide prevention media campaigns [19], limited evidence exists regarding their efficacy, in particular among young people. This study did not have the capacity to conduct a broad evaluation of the reach and acceptability of the media messages developed. As no iatrogenic effects were reported, it is possible to engage young people in the development of a suicide prevention campaign; the impact of which could then be evaluated on a larger scale.

Finally, the Facebook group proved to be a useful and safe way to communicate with young people throughout this project. This suggests that as professionals, we can be more confident in using social media platforms to communicate with young people on sensitive issues such as suicide. To the best of our knowledge, no previous studies have used social media platforms in this manner. Historically, professionals have been shown to use social media differently from young people when it comes to suicide prevention [39] and concerns exist with regard to ethical issues, including client confidentiality and duty of care [42]. Although it is right to remain cautious, particularly when considering using social media platforms in clinical care, it is becoming more apparent that these types of platforms can provide a useful and acceptable medium through which professionals can communicate with young people.

### Conclusions

Social media is increasingly being used by young people for health-related issues, including communicating about suicide [43,44]. It presents a range of benefits, including its reach, accessibility, and acceptability, and as such, it provides an ideal platform through which suicide prevention media campaigns can be delivered. This study suggests that young people can be safely engaged in the process of developing such a campaign, which as a result may have more traction with this population. Furthermore, educating young people about how to talk safely about suicide online has multiple benefits. Overall, these findings pave the way for new approaches to suicide prevention in young people.

## Appendix

Multimedia Appendix 1 Impact of student involvement questionnaire.

Multimedia Appendix 2 Media message evaluation questionnaire.

Multimedia Appendix 3 Messages developed.
