# Supplementary material for: Developing Social Media-Based Suicide Prevention Messages in Partnership With Young People: Exploratory Study
Source: JMIR Ment Health. 2017 Oct 4;4(4):e40. doi: 10.2196/mental.7847 (PMC5647460; doi:10.2196/mental.7847)
Supplement: Multimedia Appendix 1 [file mental_v4i4e40_app1.pdf]

Appendix 1: Impact of Student Involvement Questionnaire

About you

1.	What is your name?		
2.	What is your date of birth?		
3.	What is the name of your school?		
4.	What year level are you in?		
5.	What is your gender?		
6.	Have you ever experienced a mental health problem?	Yes	No
7.	Have you ever experienced suicidal thoughts or feelings?	Yes	No
8.	Have you ever hurt yourself on purpose?	Yes	No
9.	Have you ever supported a friend who was experiencing mental health problems?	Yes	No
10.	Have you ever supported a friend who was experiencing suicidal thoughts or feelings?	Yes	No
11.	Has someone you felt close to ever died by suicide?	Yes	No

Project evaluation

The next few questions will ask you what you thought about the project.

	1 Strongly disagree	2 Disagree	3 Neither agree nor disagree	4 Agree	5 Strongly agree
Participating in this program has helped me to					

develop new skills					
As a result of this program I have a better understanding of how to talk about suicide safely online					
As a result of this program I have a better understanding of how to talk about suicide generally					
The program has helped to improve my self confidence					
The program has helped me to develop my leadership and/or mentoring skills					
The program has helped me to further develop my interpersonal skills					
The program has helped me to further develop my communication skills					
As a result of participating, I feel more able to provide emotional support to others					

As a result of participating, I feel more able to educate others about cyber-safety					
The *Safe Conversations* project was enjoyable					
The *Safe Conversations* project was helpful					
The *Safe Conversations* project made me feel upset					
The *Safe Conversations* project made me feel suicidal					
The *Safe Conversations* project was boring					
The *Safe Conversations* project took up too much of my time					
I found participating in the *Safe Conversations* project stressful					
I feel motivated after participating in the *Safe Conversations* project					
The *Safe Conversations*					

project was worthwhile					

Would you recommend the *Safe Conversations* project to a friend? (*Please tick*)

Yes **No**

If you answered 'no', please tell us why….

____

____

____

____

Are there any other comments you would like to make about the project?

____

____

____

____
