# Supplementary material for: Developing Social Media-Based Suicide Prevention Messages in Partnership With Young People: Exploratory Study
Source: JMIR Ment Health. 2017 Oct 4;4(4):e40. doi: 10.2196/mental.7847 (PMC5647460; doi:10.2196/mental.7847)
Supplement: Multimedia Appendix 2 [file mental_v4i4e40_app2.pdf]

Appendix 2: Media Message Evaluation Questionnaire

About you

How old are you?	
Have you ever experienced suicidal thoughts or feelings?	❍ Yes<br>❍ No
Have you experienced suicidal thoughts or feelings in the past four weeks?	❍ Yes<br>❍ No
Are you experiencing suicidal thoughts or feelings right now?	❍ Yes<br>❍ No

Evaluation of media messages

The following set of questions was repeated for each media message.

How helpful do you think this media message will be for someone who is having thoughts of suicide?	❍ Extremely unhelpful<br>❍ Unhelpful<br>❍ Neither helpful nor unhelpful<br>❍ Helpful<br>❍ Extremely helpful
How helpful do you think this media message will be for someone who wants to help somebody else who may be having thoughts of suicide?	❍ Extremely unhelpful<br>❍ Unhelpful<br>❍ Neither helpful nor unhelpful<br>❍ Helpful<br>❍ Extremely helpful
After seeing this media message, how likely are you to seek help for suicidal thoughts or other mental health issues?	❍ More likely than before<br>❍ Less likely than before<br>❍ About the same as before

After seeing this media message, how likely are you to help somebody else who might be having suicidal thoughts or experiencing other mental health issues?	❍ More likely than before<br>❍ Less likely than before<br>❍ About the same as before
Based on the picture below, what was your mood like before seeing this media message?<br>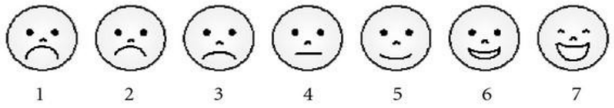	❍ 1<br>❍ 2<br>❍ 3<br>❍ 4<br>❍ 5<br>❍ 6<br>❍ 7
And what was your mood like after seeing this media message?<br>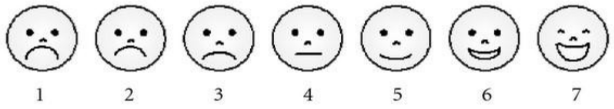	❍ 1<br>❍ 2<br>❍ 3<br>❍ 4<br>❍ 5<br>❍ 6<br>❍ 7
What did you think of the format of this media message?	❍ I really like it<br>❍ I like it<br>❍ I’m neutral<br>❍ I don’t like it<br>❍ I really don’t like it
What did you think of the content of this media message?	❍ I really like it<br>❍ I like it<br>❍ I’m neutral<br>❍ I don’t like it<br>❍ I really don’t like it
Would you share this with others?	❍ Yes<br>❍ No<br>❍ I'm not sure
Do you have any other comments about this media message?	
