# Supplementary figures and images for: Developing Social Media-Based Suicide Prevention Messages in Partnership With Young People: Exploratory Study
Source: JMIR Ment Health. 2017 Oct 4;4(4):e40. doi: 10.2196/mental.7847 (PMC5647460; doi:10.2196/mental.7847)

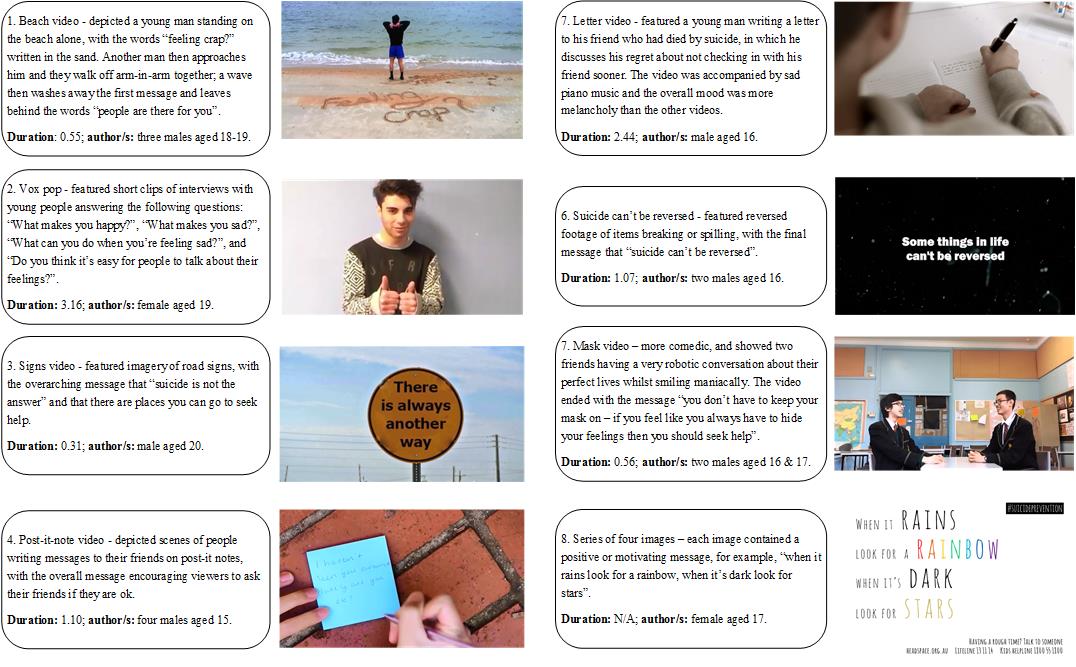

Supplement: Multimedia Appendix 3 [file mental_v4i4e40_app3.jpeg]
